# Supplementary material for: Knowledge, attitudes and practices toward Female Genital Schistosomiasis among women living in communities along the Volta Lake in Volta Region, Ghana
Source: PLoS Negl Trop Dis. 2025 Nov 3;19(11):e0013681. doi: 10.1371/journal.pntd.0013681 (PMC12599931; doi:10.1371/journal.pntd.0013681)
Supplement: S1 Table — (DOCX) [file pntd.0013681.s001.docx]

S1 Table: Knowledge of FGS among women living in communities along the Volta Lake

| **Variable** | **Frequency (N=745)** | **Percentage (%)** |
| --- | --- | --- |
| **Ever heard of FGS** |  |  |
| Yes | 314 | 42.1 |
| No | 431 | 57.9 |
| **Transmission is by contact with contaminated water** |  |  |
| Yes | 397 | 53.3 |
| No | 348 | 46.7 |
| **Know about FGS signs and symptoms** |  |  |
| Yes | 278 | 37.3 |
| No | 467 | 62.7 |
| **Know about FGS health consequences** |  |  |
| Yes | 139 | 18.7 |
| No | 606 | 81.3 |
| **FGS affects women’s quality of life** |  |  |
| Yes | 422 | 56.4 |
| No | 323 | 43.4 |
