# Supplementary material for: Knowledge, attitudes and practices toward Female Genital Schistosomiasis among women living in communities along the Volta Lake in Volta Region, Ghana
Source: PLoS Negl Trop Dis. 2025 Nov 3;19(11):e0013681. doi: 10.1371/journal.pntd.0013681 (PMC12599931; doi:10.1371/journal.pntd.0013681)
Supplement: S2 Table — (DOCX) [file pntd.0013681.s002.docx]

S2 Table: Attitude towards FGS among women living in communities along the Volta Lake

| **Variable** | **Frequency** | **Percentage** |
| --- | --- | --- |
| **FGS is not a serious health issue** |  |  |
| Yes | 338 | 45.4 |
| No | 407 | 54.6 |
| **There's the need to seek care** |  |  |
| Yes | 250 | 33.6 |
| No | 495 | 66.4 |
| **Will relate well with an FGS infected person** |  |  |
| Yes | 685 | 91.9 |
| No | 60 | 8.1 |
| **Women with FGS should be isolated** |  |  |
| Yes | 72 | 9.7 |
| No | 673 | 90.3 |
| **Willing to support an infected person** |  |  |
| Yes | 724 | 97.2 |
| No | 21 | 2.8 |
| **Willing to participate in FGS campaigns** |  |  |
| Yes | 675 | 90.6 |
| No | 70 | 9.4 |
| **Will seek care for FGS if infected** |  |  |
| Yes | 717 | 96.2 |
| No | 28 | 3.8 |
