# Supplementary material for: Knowledge, attitudes and practices toward Female Genital Schistosomiasis among women living in communities along the Volta Lake in Volta Region, Ghana
Source: PLoS Negl Trop Dis. 2025 Nov 3;19(11):e0013681. doi: 10.1371/journal.pntd.0013681 (PMC12599931; doi:10.1371/journal.pntd.0013681)
Supplement: S3 Table — (DOCX) [file pntd.0013681.s003.docx]

S3 Table: Practices towards FGS among women living in communities along the Volta Lake

| **Variable** | **Frequency** | **Percentage** |
| --- | --- | --- |
| **Ever had FGS** |  |  |
| Yes | 51 | 6.9 |
| No | 694 | 93.1 |
| **Sought care** |  |  |
| Yes | 44 | 86.7 |
| No | 7 | 13.7 |
| **Delayed in seeking care** |  |  |
| Yes | 33 | 75 |
| No | 11 | 25 |
| **Place of Seeking Care** |  |  |
| Health facility | 32 | 72.7 |
| Pharmacy/drug store | 12 | 27.3 |
| **Prompts for seeking care** |  |  |
| Health workers advice | 4 | 9.1 |
| Severity of condition | 40 | 90.9 |
| **Swim and wade in water** |  |  |
| Yes | 44 | 95.7 |
| No | 2 | 4.3 |
| **Beliefs exist about FGS** |  |  |
| Yes | 43 | 16.6 |
| No | 216 | 83.4 |
| **Cultural practices influence FGS** |  |  |
| Yes | 46 | 6.2 |
| No | 699 | 93.8 |
