# Supplementary material for: Knowledge, attitudes and practices toward Female Genital Schistosomiasis among women living in communities along the Volta Lake in Volta Region, Ghana
Source: PLoS Negl Trop Dis. 2025 Nov 3;19(11):e0013681. doi: 10.1371/journal.pntd.0013681 (PMC12599931; doi:10.1371/journal.pntd.0013681)
Supplement: Attachment — Submitted filename: Response to Reviewers.docx [file pntd.0013681.s006.docx]

15^th^ October 2025

Editor-in-Chief

PLOS Neglected Tropical Diseases

United States

Dear Dr Shaden Kamhawi

**Response to Reviewers’ Comments on Manuscript ID (PNTD-D-25-01109) – “Knowledge, attitudes, and practices toward Female Genital Schistosomiasis among women in communities along the Volta Lake in Volta Region, Ghana”**

We sincerely thank you and the reviewers for your careful evaluation of our manuscript and for the constructive comments and suggestions that have greatly improved the quality and clarity of our paper. We have revised the manuscript accordingly and provide a detailed point-by-point response below. All revisions are in track changes in the document labelled “Revised Manuscript with Track Changes”.

Below, we address each comment from the reviewers in detail in a tabular format showing the Reviewer in column 1, their comments in column 2 and our responses in column 3.

| **Reviewer** | **Comment** | **Response** |
| --- | --- | --- |
| **Reviewer 1** | **Methods** |  |
|  | Objective clear, appropriate design and analysis, no ethical concerns | We thank the reviewer for the encouraging comments regarding the clarity of the study objective, the appropriateness of the design and analysis, and the ethical rigor of the research. We are glad these aspects met the reviewer’s expectations. |
|  | **Summary and General Comments** |  |
|  | At Line 296 : ", respondents with family size >5 were times more likely ..." it should be less than 5, right ? | Thank you for drawing our attention to the error. We have corrected it to <5. This can be found on page 21 line 325 |
|  |  |  |
| **Reviewer 2** | **Methods** |  |
|  | The objectives of the study are clearly articulated, and the study design is appropriate for addressing these objectives | We sincerely thank the reviewer for the positive feedback. We are pleased that the study’s objectives, design, and analysis were found to be appropriate, and that there were no ethical concerns identified. |
|  | Data Collection Tools”  The manuscript does not sufficiently describe how the Knowledge, Attitudes, and Practices (KAP) variables were defined or validated, which affects reproducibility and reliability. The composite scores for KAP were constructed from selected questions. However, they do not reference to existing KAP frameworks for FGS or similar NTDs. Additionally, there is no justification for why certain variables were included while others were excluded. The questionnaire was adapted but does not describe (i) the source literature for the "general knowledge of FGS" questions (Line 166) and (ii) whether content validity (expert review) or internal consistency was assessed. There is no mention of how translation to local language adaptation, which is critical for KAP surveys. Minor revisions can address this. Adding 1–2 sentences on tool development and validation would strengthen the study’s credibility | Thank you for the comment. We have revised the section on Data Collection Tool to include:  (i) the reference from which the questions on general knowledge of FGS were adapted from.  (ii) We have included a statement on how the questionnaire was validated by subject knowledge experts to ensure its appropriateness. These were the supervisor of the project who is an expert in infectious and tropical diseases and the NTD focal persons of the Ghana Health Service in the study districts. We have also added the language (English) in which the questionnaire was developed and the use of trained data collectors who were fluent in English and the local languages to administer the questionnaire. These changes can be found on page 9 lines 177-188 |
|  | Data Analysis  -Line 202. Please develop a justification for the use of a p-value threshold of ≤ 0.2 for selecting variables in bivariate analysis before multivariable regression. It is unconventional and needs to be better explained | Thank you for the comment. We have provided a justification for the use p-value ≤0.2 as follows:  “This was done to avoid excluding variables that may not be significant in univariable analysis but could become significant or act as confounders once other variables were controlled for in the multivariable model”. We have provided a reference that supports this convention: Hosmer, D. W., Lemeshow, S., & Sturdivant, R. X. (2013). Applied logistic regression (3rd ed.). Wiley. This can be found on page 11 lines 220-224 |
|  | Address as limitations:  -The authors have opted to use “the bottle-spinning method: for household selection. This could introduce site-specific bias. Briefly acknowledge this in the limitations. | Thank you for the comment. We have acknowledged the use of the bottle-spinning method as a limitation as follows: “The cross-sectional design prevents causal inference and the use of the bottle spinning technique in selecting households could introduce selection bias where the households chosen along that line may not represent the entire community”…….This can be found on page 31, lines 484-486 |
|  | **Results** |  |
|  | 1. Revision of Table 1:  Improve the readability of Table 1 by clarifying income reporting. Please specify the currency conversion date (line 225: 500 ghs = 38.04 usd). Cite what type of exchange rates fluctuate (cite the source/year). Marital status. Were there no widows, or were you grouped with the divorced? Please clarify. | Thank you for your comment.  We have revised the manuscript to include the date the currency conversion was done and the source of the conversion. This can be found on page 12 line 246  As you rightly said, widows were grouped with divorced category because the numbers for the two categories were small compared to those married or single. We have revised the table to show that divorced and widowed are grouped together (Table 2). |
|  | Explain the p < 0.2 Rationale for the regression results. | Thank you for your comment. The use of p<0.2 for the regression analysis has already been explained above in response to your comment on data analysis in the methods section. The revision in the manuscript can be found on page 11 line 220-224 |
|  | Add a table on the methods that clarifies what determines what was “Good” or “poor” knowledge, attitude and practice, ideally linked to a framework and a validated tool. Help the reader and remind them in the narrative of the results. | Thank you for the comment.  We have added a table in the methods section to show the mean scores that were used in categorizing good and poor knowledge, attitude and practices (Table 1 on page 10).  The explanation for how questions were generated and validated is given on page 9, lines 176-181 |
|  | Tables and figures support the text; however, some formatting refinements are needed, especially in the tables. I recommend re-editing all of them to improve readability. Here are some suggestions: Highlight Significance: Bold significant results (e.g., p<0.05). Merge "P-Value" Column: Remove the standalone column; keep p-values with cOR/aOR. Align Reference Categories: Place "Ref" under both cOR and aOR columns for consistency. Group Non-Significant Variables: Use footnotes (for instance, "NS = Not significant in adjusted model"). | Thank you for the comment.  As suggested, all significant results have been bolded. The standalone p-value column has been removed and log likelihood p-values merged with the second column. “Ref” has been placed under both cOR and aOR for consistency and variables significant at the bivariable level but not significant at the multivariable level have been indicated with NS and explained as a footnote. Changes can be found in Table 3 on pages 16-18, Table 4 on pages 19-21 and Table 5 on pages 22-24. |
|  | Why did the authors choose not to assess differences in sites? I recommend adding site stratified results (even briefly) to strengthen the analysis. If practices vary by district, interventions should then be tailored. For instance, a particular site may need more awareness campaigns. If authors choose not to proceed with the suggestion, please add this as a limitation or need for future research. | Thank you for the comment.  We have revised the regression analysis to show stratification by sites and included that in the write up of the results. This can be found in table 3 page 18, table 4 page 21and table 5 page 24 |
|  | **Conclusions** |  |
|  | Your discussion provides a solid foundation for improving the management of schistosomiasis and FGS in Ghana. The discussion has a strong foundation but requires tighter logic, broader evidence integration, and policy relevant specifics. Please consider the following suggestions:  1. The discussion jumps between findings without a narrative. Adding subtitles to the main arguments and points of discussion that the authors wish to convey will improve readability and help organise the structure. | Thank you for this constructive feedback. We have revised the Discussion section to improve the logical flow and strengthen the linkage between study findings and their implications for schistosomiasis and FGS management in Ghana. We have also integrated additional recent literature to broaden the evidence base, ensuring that our arguments are supported by regional and global perspectives.  We have added subtitles to the main arguments and discussion points to improve readability and structure of the discussion section. The revisions on the discussion can be found on pages 24-30, lines 336-461 |
|  | The discussion fails to address how poor HCW awareness perpetuates community KAPs. HCWs are frontline educators. Their lack of training in FGS diagnosis and management directly impacts community KAP (there is a lot of studies on this). This omission undermines the call for systemic improvements. While NTD programs are mentioned, the discussion does not explicitly tie FGS to SRH services, despite its clinical overlap with gynaecological conditions. SRH platforms (like cervical cancer screening) could amplify FGS detection and education. The need for integration can be found in some key studies: (i) Integration of prevention and control measures for female genital schistosomiasis, HIV and cervical cancer. (ii) Human rights as a framework for eliminating female genital schistosomiasis. (iii) Minimum service package for the integration of Female Genital Schistosomiasis into sexual and reproductive health and rights interventions. (iv) Urogenital schistosomiasis (UGS) and female genital schistosomiasis (FGS) in Cameroon: an observational assessment of key reproductive health determinants of girls and women in the Matta Health Are. (v) “We know about schistosomiasis but we know nothing about FGS”: A qualitative assessment of knowledge gaps about female genital schistosomiasis among communities living in Schistosoma haematobium endemic districts of Zanzibar and Northwestern Tanzania. (vi) “Female genital schistosomiasis is a sexually transmitted disease”: Gaps in healthcare workers’ knowledge about female genital schistosomiasis in Tanzania. -Please consider including a list of the community KAP gaps to HCW training deficits and propose integration with SRH services. | Thank you for your comment  We acknowledge that the health care workers are frontline educators, and their lack of training affects community KAP.  We have revised the discussion to have a section on “Linking Community KAP Gaps to Healthcare Worker Knowledge Deficits” where we clearly explain how lack of knowledge of healthcare impacts KAP of the community and what can be done to improve healthcare workers’ knowledge. This can be found on page 26, lines 378-390.  We have also included a section on “Integrating FGS into Sexual and Reproductive Health Services” where we have discussed the benefits of this. This can be found on page 29, lines 451-461.  We reviewed the literature suggested and have in cooperated some of them in the discussion where necessary. |
|  | Strengthen the discussion of your findings with the previous work of Mazigo’s in Tanzania and the FAST package in Ghana and Madagascar (some were listed above). These gaps miss opportunities to align findings with proven strategies for KAP improvement. | Thank you for your comment.  We have included in our discussion the work of Mazigo and the FAST package in Ghana and Madagascar. |
|  | Strengthen your limitation chapter. Address methodological limitations like the cross-sectional design, reliance on self-reported data without triangulation of data. The lack of validation on the KAP tools. Reflect on the missed opportunities of the design of the study on examining healthcare system barriers, propose future research directions in this vein | Thank you for the comment.  We have revised the limitation section to include areas suggested by the reviewer. This can be found on page 30, lines 483-489. |
|  | Add a conclusion that summarises specific recommendations for policymakers, international organisations, and local stakeholders to support the community awareness and future research on FGS community awareness. | Thank you for the comment.  We have revised the conclusion to summarize key findings and recommendations and the need for collaboration between stakeholders at all levels to improve KAP of FGS. This can be found on page 31, lines 498-510. |
|  | **Editorial and Data Presentation Modifications** |  |
|  | Formatting: "Schistosoma haematobium" should be italicized throughout. Eliminate redundant terms (like multiple uses of "majority"). Correct: "whiles" to "while"Correct: "crudes level" to "crude level" | Thank you for the comment. We have italicized Schistosoma haematobium throughout the document, changed whiles to while and crudes to crude. We have also reduced the use of the word majority. |
|  | Citation Consistency: The manuscript currently mixes APA (author-date) and Vancouver (numbered) citation styles. Please standardize to one format throughout text and references | Thank you for drawing our attention to the inconsistent citation format.  We have formatted all in text citations and references to Vancouver style |
|  | **Summary and General Comments** |  |
|  | Reviewer #2: *Major Revision* This study provides important insights into knowledge, attitudes and practices (KAP) regarding female genital schistosomiasis (FGS) among vulnerable populations in Ghana. While the research effectively identifies critical gaps in health education, major revisions are needed to enhance its scientific rigor and policy relevance. Key areas requiring attention include: (i)methodological limitations: The use of unvalidated KAP tools and potential sampling biases should be explicitly addressed, with justification provided for the chosen methodology; (ii) the discussion should more thoroughly examine how healthcare worker knowledge gaps links with community level misunderstandings about FGS, and how this affects health seeking behaviors; (iii) the paper would benefit from specific recommendations on integrating FGS education into existing sexual and reproductive health services; (iv) claims should be supported by appropriate references to comparable studies in similar settings, with particular attention to West African research on neglected tropical diseases. | Thank you for the comments. We have addressed all the comments raised here in the previous comments made by the reviewer as outlined above. |
|  | Introduction Lines 81-82): "The statement '112 million people worldwide are infected with FGS' requires clarification. Given limited global burden studies, I recommend revising to: 'An estimated 56 million women in sub-Saharan Africa currently suffer from FGS (1).' This maintains accuracy while reflecting available evidence." Lines 91-92: you state that “The problem is further worsened by social stigma and destruction of the hymen, leading to accusations of sexual promiscuity”. This claim needs stronger evidence. Reference 6 (Kukula) doesn't support this specific claim. Please either remove this statement, or cite a more appropriate reference documenting this phenomenon. | Thank you for the comment. We have revised the sentence as suggested and deleted the other sentence as advised. The changes can be found on page 4 line 84 |
| Reviewer 3 | The manuscript discusses a significant and relevant subject on neglected tropical diseases (NTDs). However, it needs to be revised before acceptance. Please consider the following comments to improve the quality of the manuscript | We sincerely thank the reviewer for their careful evaluation of our manuscript and for recognizing the importance of our work on neglected tropical diseases (NTDs). We appreciate the insightful comments and suggestions, which have been invaluable in improving the clarity and overall quality of the paper. |
|  | (Sampling Method) How many districts on the Volta region? Why were these three districts chosen based on what specific criteria? It specifies that the first household was sampled using a bottle spin, but not how other households were selected | Thank you for your comment. We have indicated the number of districts in the Volta region and the reason why the three districts were selected as follows: “A multi-stage sampling technique was used to select females from three districts out of 18 districts in the Volta region. The three districts were purposively selected because of the Volta Lake passing through them exposing communities along its banks to the risk of FGS”. This can be found on page 8, lines 158-160  We have also revised the manuscript to explain how subsequent households were selected after the first household as follow: “Households within the communities were selected by locating the center of the community and spinning a bottle to identify the starting cluster. The nearest house in that cluster was selected to start the survey. After conducting interviews in the first house, moving in the right direction, two houses were skipped and the third house selected as the next household. This procedure was repeated till the survey was completed in that community” This can be found on page 8, lines 164-167 |
|  | (Discussion) • In the discussion section, there are statements about KAP (e.g. "knowledge is affected by attitude and occupation," "the education increases the good attitude by 2.6 times") without having presented any of the required statistical evidence to support them. For all such claims, the manuscript must contain the estimates of the association (e.g., adjusted odds ratio) and 95% confidence intervals, and P-values from multivariable logistic analysis. This information would allow readers to assess the relationship. • The authors used different reference styles in the discussion. | Thank you for your comment.  In response to a comment by reviewer 2, we have revised the entire discussion section which no longer includes the quoting of the odds ratios. The revised discussion can be found on pages 24-30 lines 336-461  We have also revised the referencing style to include only Vancouver style. |

Thank you for your consideration of our manuscript.

Yours sincerely

Joyce B. Der

Corresponding author
